# Editorial: Contemporary Models in Ectodermal Organ Development, Maintenance and Regeneration

**DOI:** 10.3389/fphys.2021.758271

**Published:** 2021-09-24

**Authors:** Abigail Saffron Tucker, Maisa Hanna-Maija Seppala

**Affiliations:** Centre for Craniofacial and Regenerative Biology, King's College London, Guy's Hospital, London, United Kingdom

**Keywords:** ectodermal organ, development, tooth, gland, palate, tongue

This Research Topic stems from the 2019 Tooth Morphogenesis and Development (TMD) Meeting held in Oxford, UK. The topic expands the interactions and discussions initiated during the meeting, and acts as a snapshot of the current research in ectodermal organs, particularly those of the head. Ectodermal organs, include tooth, glands, and hair, are essential for physiological functions such as feeding, maintenance of normal body temperature and homeostasis of microenvironments. Development of these organs is extensively studied and forms the basis of many ectodermal organ regeneration strategies. The topic includes 14 papers focused on ectodermal organ development in the craniofacial region, and the impact of defects in development on these structures. The papers straddle three main areas: tooth/oral, palate, and gland development.

Papers on tooth development spanned early epithelial thickening to later differentiation and maturation of the dental tissues (Figure 1). Qiu et al. investigate the development of the dental lamina (DL) during human embryonic and foetal development, focusing on its association with the adjacent vestibular lamina (VL). The paper highlights the close relationship of the VL and DL and confirms in culture the linked origin of the two laminas in the lower incisor region. The paper then goes on to look at the mechanisms used to generate the oral vestibule (cheek/lip furrow) by a process involving epithelial differentiation, differential proliferation and ultimately cell death to create the furrow. Ultimately this research can be used to understand the mechanisms underlying defects in vestibule formation, as observed in syndromes such as Ellis-van Creveld syndrome.

**Figure 1:**
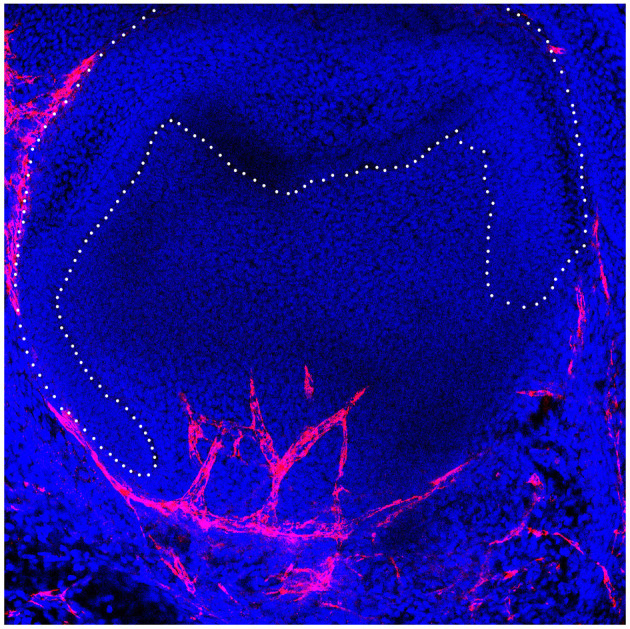
Developing murine tooth germ with invading vasculature in red.

Sánchez et al. move to morphogenesis of the tooth and highlight the role of hyaluronan in controlling both tooth shape and number, suggesting a trade-off between the two. While the function of proteins has been widely studied in the tooth, very little research has looked at the role of carbohydrates. Hyaluronan is an important structural component of the extracellular matrix and has been shown to coordinate processes such as cell shape, proliferation and migration in other organs. Here, Sanchez shows that inhibition of HA synthesis in the mouse molar led to changes in cell orientation and shape, leading to larger but fewer molars. Carbohydrates can therefore provide instructive information to control important cell decisions.

Keeping to the theme of tooth number, Brink et al. investigate whether removal of a replacing tooth (second generation) impacted formation of the rest of the dental lamina and the stem cell population that has been hypothesised to reside there. For this they utilised the polyphodont (continuously replacing) gecko as a model. The dental lamina holds the generations of replacement teeth together, like beads on a necklace. Surgical removal of the second unerupted tooth, therefore, disrupted this link. Calcein and Xylenol orange dyes were used to chart the return of replacement teeth, while BrDU pulse chase experiments, followed the location of label retaining cells in the lamina. Interestingly they show that tooth replacement patterns recovered within 3 months, highlighting the regenerative potential of the gecko dentition. Overall, the study confirms that the dental lamina contains a source of dental stem cells but also that this structure contains the information to correctly pattern newly initiating teeth.

Said et al. investigate the role of the circadian clock in regulation of enamel thickness and mineralisation focusing on the connexion between amelogenesis and calcium signalling. Individuals with mutations in STIM 1, a component of the store operated calcium entry (SOCE) signalling pathway, have hypomineralised enamel characterised as amelogenesis imperfecta (AI). To understand the underlying mechanisms, the authors showed that loss of STIM1 specifically in ameloblasts in conditional knockout mice led to changes in circadian activator and inhibitor genes. These changes in the circadian clock were hypothesised to contribute to the enamel defects observed in STIM1-mediated AI.

Keeping with ameloblasts, Ruspita et al. investigated the control of amelogenesis by the *Msx2-Sp6-Follistain* pathway. Mutations in either *MSX2* or *SP6* lead to AI, with mutant mice showing similar defects in enamel. Here the authors show that these genes work in the same pathway, with overlapping expression in secretory ameloblasts, and both inhibiting *follistatin* expression in dental epithelial cell lines. Transient transfection of *Msx2* lead to an upregulation of *Sp6* and downregulation of *Follistatin*, with ChIP confirming the binding of Msx2 to the *Sp6* promotor. Together these experiments unpick the complex transcriptional networks at play during amelogenesis and help to further explain the mechanisms underlying AI.

Malik et al. turn to mineralisation of the tooth and the interplay between morphogenesis and differentiation. Here they show that Bone morphogenetic protein 7 (Bmp7) was important for controlling the onset of mineralisation in the mouse molar, with a delay in mineralisation observed in conditional mutants at birth. Unlike *Bmp2*, which is known to affect odontoblast polarity, loss of *Bmp7* in the neural crest derived mesenchyme led to a loss of Wnt signalling in the overlying pre-ameloblasts. *Bmp6* was also upregulated, suggesting some compensation mechanisms at play. The delay in initiation affected the shape of the final cusps, linking changes in differentiation to changes in shape.

On the clinical side, the link between Wnt signalling and non-syndromic tooth agenesis (NSTA) was further strengthened by analysis of Thai individuals by Kanchanasevee et al.. Tooth agenesis is one of the most common orodental anomalies, and mutations in *WNT10A* and the *EDA* signalling pathway have previously been highlighted as causing defects in early tooth development. Here novel variants in both *WNT10a* and *EDARADD*, the intracellular adapter for the *EDA* pathway, were discovered, enhancing our knowledge of how specific variants can impact tooth development.

The Eda pathway was also studied by Jia et al. who investigated the interaction between *Eda* and *Pax9*. Both genes in patients cause selective tooth agenesis, and here the authors show that compound mouse mutants have specific loss of the lower incisors, with higher incidence of loss of the third molar. Loss of the incisors was associated with downregulation of *Fibroblast growth factor (Fgf)3* and *Sonic Hedgehog* signalling, with defects evident from the bud to cap stage and arrest thereafter. Overall, this paper highlights the interaction between *Eda* and *Pax9*, working together to control odontogenesis.

Continuing the theme of Wnt signalling in ectodermal organs, Eliason et al. investigate the interaction between *Lef1*, a key downstream readout of canonical Wnt signalling and the microRNA miR-26b. Overexpression of miR-26b mimicked the ectodermal phenotype of *Lef1* null mice, while it rescued the defects in *Lef1* overexpression mice. This paper shows the key role that microRNAs play in regulating signalling pathways, and, in the case of the tooth, in regulating the stem cell niche.

Jumping from the tooth to the neighbouring tongue, Zhang et al. investigate the formation of the circumvallate papilla (CVP). This papilla, found at the back of the tongue, houses the von Ebner's glands, and has a high density of tastebuds, and complex folded shape. How this shape is created from a placodal thickening was investigated by looking at the dynamics of actomyosin dependent constriction. Using an elegant explant culture system, they show that morphogenesis is driven by epithelial cell constriction, dependent on focal adhesion kinase, with *Shh* signalling coordinating the process. Recombination between epithelium and mesenchyme tongue tissue highlighted the role of *Fgf10* in driving the invagination of the CVP epithelium.

The creation of complex shapes from simple epithelial thickenings at the cell level was a central focus of the review from Mogollón and Ahtiainen. Here they follow how live imaging can be used to track cell level decisions, and the latest advances in technology and culture that allow resolution at the cell level. These include the use of fluorescent reporter mice combined with confocal microscopy and highlight how live imaging advances in hair follicles have been recently applied to tooth placodes, with promise for use in other ectodermal organs.

Two papers in this Research Topic shed light on the delicate mechanisms of primary and secondary palate fusion and disappearance of the epithelial palatal seam. Yamamoto et al. explore the previously less well-known mechanisms of primary and secondary palate fusion and specifically observed the epithelial cell behaviours using palatal explant cultures of K-14-GFP mice. SEM analysis of time-lapse observations of these mice revealed that some of the epithelial behaviours of the primary palate, such as rostral extrusion and apoptosis in the presumptive fusion site with the secondary palate, happen independently without any contact with the secondary palate. In contrast, they did not identify any cellular migration that results in mesenchymal exposure during secondary palate development.

Ohki et al. investigate the molecular control of soft palate development and identified that TGF-β and SHH regulate expression of the extracellular matrix glycoprotein, Tenascin-C (TNC). TNC is required for morphological changes of the palatal shelves and its unique expression in the posterior part of the palatal shelves appeared to rely specifically on the epithelial activity of Tgf-β. Agreeing with this, TNC levels were diminished when TGF-β type II receptor was conditionally removed from the palatal epithelial cells. SHH also increased the expression of TNC mRNA and protein in palatal mesenchymal cells, providing further understanding of the aetiology of facial clefts.

Finally, moving to the eye, Liu et al. investigate the molecular changes associated with primary dry eye in the Aquaporin 5 null mouse. These mice develop spontaneous dry eye due to defects in the epithelial cells of the lacrimal gland. Using a transcriptomic approach, the authors identified molecular changes in the mutant glands, focusing on differentially expressed circular RNAs. The molecular changes highlighted a potential role for phagosomes in the pathogenesis of dry eye disease, suggesting new treatment options for this condition.

Overall, this Research Topic highlighted the flexible approaches used to understand ectodermal organ development, providing mechanistic insight into how defects in ectodermal organs occur and suggesting novel avenues for future treatments.

## Author Contributions

All authors listed have made a substantial, direct and intellectual contribution to the work, and approved it for publication.

## Conflict of Interest

The authors declare that the research was conducted in the absence of any commercial or financial relationships that could be construed as a potential conflict of interest.

## Publisher's Note

All claims expressed in this article are solely those of the authors and do not necessarily represent those of their affiliated organizations, or those of the publisher, the editors and the reviewers. Any product that may be evaluated in this article, or claim that may be made by its manufacturer, is not guaranteed or endorsed by the publisher.

